# Supplementary material for: Hybrid Models and Biological Model Reduction with PyDSTool
Source: PLoS Comput Biol. 2012 Aug 9;8(8):e1002628. doi: 10.1371/journal.pcbi.1002628 (PMC3415397; doi:10.1371/journal.pcbi.1002628)
Supplement: Text S4 — Complete source code for the PyDSTool package (version 0.88.120504). Includes API documentation and help files linking to web pages. This file is identical to the current public release on Sourceforge.net. (ZIP) [file pcbi.1002628.s004.zip › PyDSTool/html/toc-PyDSTool.Events-module.html]

xml version="1.0" encoding="ascii"?


Events


# Module Events

---

## Classes

Event  
 EventStruct  
 HighLevelEvent  
 LowLevelEvent  
 MatlabEvent  

## Functions

\_active

\_highlevel

\_lowlevel

\_nonterm

\_notactive

\_notprecise

\_notvarlinked

\_precise

\_term

\_varlinked

collectReused

findpreciseroot

makePythonStateZeroCrossEvent  
 makeZeroCrossEvent  

processReusedC

processReusedMatlab

processReusedPy

## Variables

\_1DimplicitSolveMethods

\_all\_complex

\_all\_float

\_all\_int

\_all\_numpy\_complex

\_all\_numpy\_float

\_all\_numpy\_int

\_complex\_types

\_float\_types

\_implicitSolveMethods

\_indentstr

\_int\_types

\_num\_equivtype

\_num\_maxmin

\_num\_name2equivtypes

\_num\_name2type

\_num\_type2name

\_num\_types

\_pytypefromtype

\_real\_types

\_seq\_types

---

[hide private]
